# Recent Advances in Breast Cancer Diagnosis, Treatment, Psychology, Management, and Reconstruction

**DOI:** 10.3390/medicina59020212

**Published:** 2023-01-22

**Authors:** Jennifer Den, Andrea Sisti

**Affiliations:** 1 Department of Surgery, Division of General Surgery, University of Texas Medical Branch, Galveston, TX 77550, USA; 2 Shriners Hospital for Children, 815 Market Street, Galveston, TX 77550, USA

Breast cancer is the second most common cancer in women after skin cancer [1]. New advances in technology and management options continue to be described, allowing for an early diagnosis and a wide array of reconstruction techniques and treatments. This Special Issue [2] of *Medicina*, “Recent Advances in Breast Cancer Diagnosis, Treatment, Psychology, Management, and Reconstruction”, includes six articles that highlight new innovations with regard to breast cancer, including reconstruction and treatment options, socioeconomics and psychological issues among breast cancer patients, and an uncommonly described type of breast cancer.

It is well known that the socioeconomic status of all cancer patients contributes to the availability of care and time of diagnosis, with many of these patients less likely to have the resources for regular screening. The study provided by Jensen et al. [3] examined a population in West Texas and suggested considering earlier screening for breast cancer in this population, as well as for others with similar sociodemographic determinants. The study by Kazlauskiene et al. [4] further emphasized the importance of paying attention to the psychological aspect of breast cancer and how this diagnosis may increase suicidal ideation. Not only should physicians recognize elements that may delay cancer diagnosis, but we should also strengthen communication skills and recognize additional risk factors for psychological stressors.

This Special Issue of *Medicina* also showcases the considerable progress and continued innovation in oncoplastic reconstruction. Numerous techniques including autologous and implant reconstructions are available and widely used. The study by Sisti et al. [5] offers a reconstruction option using a superior anterior biological acellular dermal matrix and an inferior anterior dermal sling support. This particular option allows for a single-stage procedure as well as lowered costs. Treatment options have also continued to improve with the use of immunotherapy in combination with cytotoxic chemotherapy. However, new discoveries have identified that this combination may not be beneficial in patients with early triple-negative breast cancer, as discussed in the study by Rizzo et al. Furthermore, the use of photodynamic therapy also demonstrates a possible alternative treatment option (Ostanska et al. [6]). These studies emphasize the continued search for new solutions that optimize breast cancer treatment, prevent undesirable outcomes, and support patient satisfaction and well-being. 

Finally, the case report by Nanev et al. [7] shows that despite all the advances in breast cancer research, there is still much to be discovered. Cases of lymphoepithelioma-like breast carcinoma (LELC) can be found in the literature, but there has not been a clearly defined treatment, much less an option for LELC occurring simultaneously with other carcinomas of the breast as is described in this case report.

The papers published in this Special Issue showcase the continued need to approach breast cancer in a multidisciplinary fashion. The collaborative contributions reinforce that there is much to learn about the scientific and social aspects that affect breast cancer.
